# Submesoscale daily data from a non-hydrostatic OGCM at 1/90° resolution over Northern South China Sea in 2019

**DOI:** 10.1038/s41597-026-06653-1

**Published:** 2026-01-24

**Authors:** Zhanpeng Zhuang, Zhenya Song, Qi Shu, Lina Sun, Yeli Yuan

**Affiliations:** 1 First Institute of Oceanography, and Key Laboratory of Marine Science and Numerical Modeling, Ministry of Natural Resources, Qingdao, 266061 China; 2 Laboratory for Regional Oceanography and Numerical Modeling, Qingdao Marine Science and Technology Center, Qingdao, 266237 China; 3 National Engineering Laboratory for Integrated Aero-Space-Ground-Ocean Big Data Application Technology, Xi’an, 710072 China

**Keywords:** Physical oceanography, Hydrology, Physical oceanography

## Abstract

Numerical simulations of oceanic variations at submesoscale spatial scales are essential for understanding dynamic characteristics of the Northern South China Sea (NSCS). Currently, the spatial resolution and accuracy of available reanalysis and simulation datasets are still inadequate to comprehensively capture submesoscale processes, leading to an incomplete depiction of the region’s detailed dynamic characteristics. In this study, a regional oceanic simulation dataset at (1/90) ° × (1/90) ° spatial and daily temporal resolutions for the year 2019 is produced based on a non-hydrostatic ocean general circulation model (OGCM). Assessments from an idealized experiment suggest that non-hydrostatic calculations can improve the simulation accuracy of high-resolution OGCMs for small-scale oceanic features, such as internal tides or submesoscale phenomena. Comparisons with the observations demonstrate that simulations from the non-hydrostatic OGCM are generally more accurate than those from hydrostatic OGCM. Along with its good performance in simulating dynamic processes in the NSCS, this dataset can enhance understanding of the dynamical patterns and interactions among multi-scale processes, including large-scale circulation, mesoscale eddies, and submesoscale phenomena.

## Background & Summary

As the largest semi-closed marginal sea in the western Pacific Ocean, the South China Sea (SCS) is abundant with multi-scale dynamical processes, especially in its northern regions. The dynamic patterns of the Northern SCS (NSCS) are influenced by the topography and shorelines around the Luzon Strait (LS), as well as the intrusion of the Kuroshio^1–4^. In the NSCS, strong diurnal and semi-diurnal internal tides (ITs) are generated at the LS due to tidal propagation and interactions with the ridges, then propagate westward into the SCS^5–7^. The mesoscale eddies and the associated submesoscale filaments, fronts, and small-scale turbulence play an important role in the variability of the thermodynamic structure and the mass transports^8–11^. The Kuroshio flows northward to the east of Luzon Island and intrudes into the SCS through the LS. Due to the complex interactions among multiple oceanic scales, the NSCS can be regarded as a typical and natural area for testing the abilities of the non-hydrostatic ocean general circulation models (OGCMs). With advances in observation technology and modeling capabilities over the past decade, the interactions among large-scale circulations, mesoscale and submesoscale eddies, and small-scale internal waves in the NSCS have been extensively studied^12,13^.

High-resolution numerical simulations of OGCMs are important data sources for research on the dynamical processes in the NSCS. Compared with satellite datasets and *in-situ* observations, one of the advantages of OGCMs is their ability to simulate the three-dimensional structure of the ocean, combined with the flexibility to achieve high temporal resolution in model results. When the resolution increases to capture the submesoscales, thermal fronts, secondary circulation, and horizontal and vertical transport are strengthened^14,15^. However, the hydrostatic approximation should be revoked in order to address more accurately the small-scale dynamic processes and non-hydrostatic component of the pressure gradient^16–18^. The hydrostatic balance becomes invalid when the horizontal scales of the motions are comparable to the local vertical scales in some regional or coastal simulations, such as ITs over the continental shelf edge and ocean ridges, as well as small-scale processes in narrow strait passages^19–22^. The non-hydrostatic processes contribute to generating strong vertical transport^23,24^.

To develop accurate and stable non-hydrostatic OGCMs, various numerical methods have been used. The primary concern for the non-hydrostatic algorithms is the treatment of the hydrostatic and non-hydrostatic pressure. Chorin^25^ and Kim and Moin^26^ introduced the classical “projection” method,

1 $${u}^{n+1}={u}^{\ast }-\Delta t\cdot G\phi ,$$

where *u* denotes the velocity, Δ*t* is the time step, *G* is the discrete gradient operator, and *ϕ* can be regarded as an orthogonal projection of velocity. This method is inherently only first-order in time due to commutation errors, and achieving second- or third-order accuracy requires extensive multi-step iteration^27,28^. Alternatively, the “pressure correction” (PC) method can be used with second-order accuracy,

2 $${p}^{n+1}={p}^{n}+{p}^{\ast }-\Delta tL{p}^{\ast },$$

where *p* is the pressure, p^*^ is the pressure correction, and *L* is the discrete Laplace operator. In the PC method, the velocity is first solved, then the pressure is adjusted, and the non-hydrostatic pressure increment and surface elevation are updated^29,30^. In terms of time accuracy and computational cost, the PC method generally outperforms the projection method^27^, and it has been widely used to develop non-hydrostatic OGCMs^18,21,31,32^. Overall, the PC method demonstrates stronger stability and scalability compared to the projection method and explicit schemes, and has been more widely adopted in current international non-hydrostatic OGCMs. The PC method has been used to implement non-hydrostatic calculations in an independently developed Marine Science and Numerical Modelling (MASNUM) OGCM.

In the LS and its adjacent regions in the NSCS, ITs are generated and evolve subsequently into internal solitary waves (ISWs), which are induced by the interaction of the barotropic tide-induced background current field with large topographic variations and stratification. Furthermore, submesoscales can result in strong vertical velocities and tracer transport because the geostrophic balance is broken. Significant vertical motions are excited during these evolution processes. Non-hydrostatic models should offer superior capability in capturing such vertical motions and transport. This paper presents a submesoscale-resolving simulation at (1/90) ° × (1/90) ° spatial resolution over the NSCS in 2019 based on the non-hydrostatic version of MASNUM OGCM. Preliminary evaluation from an idealized experiment for standing waves in a closed basin demonstrates that the non-hydrostatic OGCM can capture the long-term variation in the current field. Further assessment of the datasets was conducted through comparative analysis with the observations, including the Moderate-resolution Imaging Spectroradiometer (MODIS) images, Optimum Interpolation Sea Surface Temperature (OISST) products, and Argo profiles. The comparisons of the IT patterns reveal that the non-hydrostatic OGCM yield stronger vertical velocities. Additionally, both the temperature profile and sea surface temperature (SST) simulations of the non-hydrostatic OGCM are closer to the Argo data and OISST than those of the hydrostatic OGCM. The datasets can contribute to a more accurate understanding of the dynamical characteristics and seasonal variations of mesoscale eddies and sub-mesoscale processes in the NSCS.

## Methods

### Model and its configuration

The non-hydrostatic version of the MASNUM OGCM^33–35^ is used for high-resolution numerical simulations. The PC method is implemented for non-hydrostatic dynamics and computations. The horizontal grids of the MASNUM OGCM are the Arakawa C-grids, which are widely used in OGCMs. The two-time-level Eulerian forward-backward (FB) scheme is employed to build a numerical solution framework in the MASNUM OGCM^33^. The two-level finite difference scheme is naturally free of the computational mode, which is generated by the time-splitting instability for almost all three-level schemes^36^. To suppress high-frequency noise and address the computational mode, the Robert-Asselin time filter algorithm^37,38^ is adopted into the three-level schemes, although this filter degrades the global truncation accuracy from second order to first order^36^. The discretized non-hydrostatic pressure gradient terms are incorporated into the FB scheme to calculate the velocity field, with an increment of the non-hydrostatic pressure derived from solving a Poisson system. σ coordinates is used in the MASNUM OGCM to represent the free surface and bathymetry. Previous studies have validated non-hydrostatic modelling through ideal or analytical tests^18,39,40^, but there has been limited validation of its performance in real-world conditions. Therefore, the necessary further investigations are conducted in this study.

Calculating non-hydrostatic pressure or momentum is crucial for implementing non-hydrostatic OGCMs. In the projection method, provisional velocities are calculated from the incomplete momentum equations without the pressure gradient terms at first, and then the velocities are modified by a variable *ϕ* to satisfy the continuity equation^25,26^. *ϕ* can be directly obtained by solving a discrete Laplace equation. The adjustment, known as the projection algorithm, does not alter the vorticity. The process involves time-stepped steps to separate hydrostatic and non-hydrostatic velocity components. However, the time accuracy of the projection algorithm is first order with standard boundary conditions and implicit discretization for the viscous terms^27^.

**Fig. 1:**
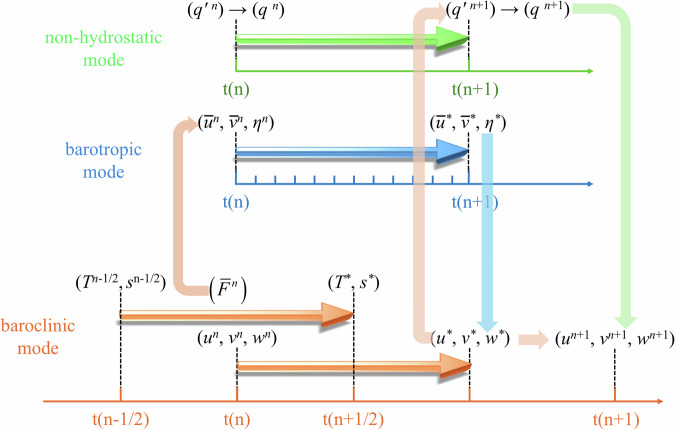
Schematic representation of the numerical algorithm system, including baroclinic mode (internal mode, orange), barotropic mode (external mode, blue) and non-hydrostatic mode (green), adopted in MASNUM OGCM. The arrows with shadows represent one integration of the numerical kernels, the pure-color arrows stand for the variable exchange among the modes.

Unlike the projection algorithm, the PC method has been widely used in non-hydrostatic OGCMs^17,21,41^. Mahadevan *et al*.^42^ and Armfield and Street^27^ demonstrated that can offer advantages over the projection method due to its second-order time accuracy and more efficient computation. In this study, the (PC) algorithm divides the total pressure into hydrostatic and non-hydrostatic components for the non-hydrostatic MASNUM OGCM (MASNUM-NonHydro). As shown in Fig. 1, the provisional barotropic two-dimensional velocities and surface elevation are obtained by solving two-dimensional barotropic equations. Following Blumberg and Mellor^43^ and Durran^36^, a mode splitting method is used, incorporating the difference in advection and diffusion terms of the barotropic velocities and their vertical integration in the baroclinic equations is added into the barotropic equations. This ensures timely updates of advection and diffusion processes and constrains barotropic velocities by both hydrostatic and non-hydrostatic components. Next, the provisional velocities, temperature, and salinity are calculated by numerically solving the governing equations based on the FB finite difference scheme, which can be regarded as a temporal staggering method. In the FB scheme, the velocities *u*, *v*, and *w* are updated at time levels *n*, *n* + 1, and so on, while the tracers *T* and *s* are updated at time levels *n* + 1/2, *n* + 3/2, and so on (Fig. 1). The FB scheme is second-order time accuracy that is equivalent to the three-level schemes like the leapfrog scheme, but more stable and more efficient than the three-level schemes because it is free of undamped computational mode and less computational cost^33^. Finally, the baroclinic velocities are updated by the incremental velocities *u*′, *v*′, and *w*′, calculated from the Poisson equations with the incremental non-hydrostatic pressure *q*′.

### Numerical implementation

Under the Boussinesq approximation, the non-hydrostatic momentum governing equations in *σ* coordinates are expressed as

3 $$\begin{array}{c}\frac{\partial (Du)}{\partial t}+\frac{\partial (D{u}^{2})}{\partial {x}_{1}}+\frac{\partial (Duv)}{\partial {x}_{2}}+\frac{\partial (u\omega )}{\partial \sigma }-fvD=-\frac{1}{{\rho }_{0}}\left(\frac{\partial p}{\partial {x}_{1}}-{A}_{{x}_{1}}\frac{\partial p}{\partial \sigma }\right)\\ +\frac{1}{D}\frac{\partial }{\partial \sigma }\left({K}_{M}\frac{\partial u}{\partial \sigma }\right)+D\frac{\partial }{\partial {x}_{1}}\left(2{A}_{M}\frac{\partial u}{\partial {x}_{1}}\right)+D\frac{\partial }{\partial {x}_{2}}\left[{A}_{M}\left(\frac{\partial v}{\partial {x}_{1}}+\frac{\partial u}{\partial {x}_{2}}\right)\right],\end{array}$$

4 $$\begin{array}{c}\frac{\partial (Dv)}{\partial t}+\frac{\partial (Dvu)}{\partial {x}_{1}}+\frac{\partial (D{v}^{2})}{\partial {x}_{2}}+\frac{\partial (Dv\omega )}{\partial \sigma }+fuD=-\frac{1}{{\rho }_{0}}\left(\frac{\partial p}{\partial {x}_{2}}-{A}_{{x}_{2}}\frac{\partial p}{\partial \sigma }\right)\\ +\frac{1}{D}\frac{\partial }{\partial \sigma }\left({K}_{M}\frac{\partial v}{\partial \sigma }\right)+D\frac{\partial }{\partial {x}_{1}}\left[{A}_{M}\left(\frac{\partial v}{\partial {x}_{1}}+\frac{\partial u}{\partial {x}_{2}}\right)\right]+D\frac{\partial }{\partial {x}_{2}}\left(2{A}_{M}\frac{\partial v}{\partial {x}_{2}}\right),\end{array}$$

5 $$\begin{array}{c}\frac{\partial (Dw)}{\partial t}+\frac{\partial (Dwu)}{\partial {x}_{1}}+\frac{\partial (Dwv)}{\partial {x}_{2}}+\frac{\partial (w\omega )}{\partial \sigma }=-\frac{\rho }{{\rho }_{0}}gD-\frac{1}{{\rho }_{0}}\frac{\partial p}{\partial \sigma }\\ +\frac{1}{D}\frac{\partial }{\partial \sigma }\left({K}_{M}\frac{\partial w}{\partial \sigma }\right)+D\frac{\partial }{\partial {x}_{1}}\left(2{A}_{M}\frac{\partial w}{\partial {x}_{1}}\right)+D\frac{\partial }{\partial {x}_{2}}\left(2{A}_{M}\frac{\partial w}{\partial {x}_{2}}\right),\end{array}$$

where *x*_1_ and *x*_2_ are the Cartesian coordinates in the zonal and meridional directions, respectively. *u* and *v* are the horizontal components of the velocity, and *ω* is the vertical velocity transformed into the σ coordinates. *D* = *H* + *η* is the height of the water column, where *H* and *η* denote the static depth and free surface elevation, respectively. *g* represents the gravitational acceleration. *ρ* and *ρ*_0_ are the water density and the reference density, respectively. *f* is the Coriolis parameter. *A*_*M*_ and *K*_*M*_ are the horizontal viscosity and vertical eddy viscosity, respectively. ${A}_{{x}_{i}}=\frac{\partial \sigma }{\partial {x}_{i}}=\frac{1}{D}\left[(1+\sigma )\frac{\partial \eta }{\partial {x}_{i}}+\sigma \frac{\partial H}{\partial {x}_{i}}\right]$, where *i* is chosen as 1 and 2. In *σ* coordinates, the transformed vertical velocity *ω*, which is perpendicular to the *σ* planes, can be written using a σ-coordinate transformation as

6 $$\omega =D\frac{d\sigma }{dt}=D\cdot \left(\frac{\partial \sigma }{\partial t}+u\frac{\partial \sigma }{\partial {x}_{1}}+v\frac{\partial \sigma }{\partial {x}_{2}}+w\frac{\partial \sigma }{\partial {x}_{3}}\right)$$

where *w* is the vertical velocity at the *Z* coordinates.

To improve the efficiency, the hydrostatic model computation is separated into two-dimensional external (barotropic) mode with small time step and three-dimensional internal (baroclinic) mode with large time step^44^. Sea surface height, *η*, and barotropic velocities are calculated by numerically solving the barotropic mode equations.

The provisional velocities are denoted as u^*^, v^*^, and w^*^, respectively. The discretized momentum equations are expressed as

7 $$\begin{array}{c}\frac{{u}_{i,j,k}^{\ast }{D}_{i,j}^{\ast }-{u}_{i,j,k}^{n}{D}_{i,j}^{n}}{\Delta t}=-{{\rm{ {\mathcal F} }}}_{1}^{n}-\frac{{D}_{i,j}^{n}}{2{\rho }_{0}}(\begin{array}{c}{{\delta }_{x}{q}^{n-1/2}|}_{i,j,k}+{{\delta }_{x}{q}^{n-1/2}|}_{i-1,j,k}+\\ {{\delta }_{x}\sigma |}_{i,j,k}{\delta }_{\sigma }{q}^{n-1/2}+{{\delta }_{x}\sigma |}_{i-1,j,k}{\delta }_{\sigma }{q}^{n-1/2}\end{array})\\ +\frac{1}{2{D}_{i,j}^{n}}\left\{{{\delta }_{\sigma }\left[\frac{{K}_{M}}{2}({{\delta }_{\sigma }{u}^{\ast }|}_{i,j,k}+{{\delta }_{\sigma }{u}^{\ast }|}_{i,j,k-1})\right]|}_{i,j,k}+{{\delta }_{\sigma }\left[\frac{{K}_{M}}{2}({{\delta }_{\sigma }{u}^{\ast }|}_{i,j,k}+{{\delta }_{\sigma }{u}^{\ast }|}_{i,j,k-1})\right]|}_{i,j,k-1}\right\},\end{array}$$

8 $$\begin{array}{c}\frac{{v}_{i,j,k}^{\ast }{D}_{i,j}^{\ast }-{v}_{i,j,k}^{n}{D}_{i,j}^{n}}{\Delta t}=-{{\rm{ {\mathcal F} }}}_{2}^{n}-\frac{{D}_{i,j}^{n}}{2{\rho }_{0}}(\begin{array}{c}{{\delta }_{y}{q}^{n-1/2}|}_{i,j,k}+{{\delta }_{y}{q}^{n-1/2}|}_{i,j-1,k}+\\ {{\delta }_{y}\sigma |}_{i,j,k}{\delta }_{\sigma }{q}^{n-1/2}+{{\delta }_{y}\sigma |}_{i,j-1,k}{\delta }_{\sigma }{q}^{n-1/2}\end{array})\\ +\frac{1}{2{D}_{i,j}^{n}}\{{{\delta }_{\sigma }[2{K}_{M}({{\delta }_{\sigma }{v}^{\ast }|}_{i,j,k}+{{\delta }_{\sigma }{v}^{\ast }|}_{i,j,k-1})]|}_{i,j,k}+{{\delta }_{\sigma }[2{K}_{M}({{\delta }_{\sigma }{v}^{\ast }|}_{i,j,k}+{{\delta }_{\sigma }{v}^{\ast }|}_{i,j,k-1})]|}_{i,j,k-1}\},\end{array}$$

9 $$\frac{{w}_{i,j,k}^{\ast }{D}_{i,j}^{\ast }-{w}_{i,j,k}^{n}{D}_{i,j}^{n}}{\Delta t}=-{{\rm{ {\mathcal F} }}}_{3}^{n}-\frac{1}{2{\rho }_{0}}({{\delta }_{\sigma }{q}^{n-1/2}|}_{i,j,k}+{{\delta }_{\sigma }{q}^{n-1/2}|}_{i,j,k-1})+\frac{1}{{D}_{i,j}^{n}}{\delta }_{\sigma }({K}_{M}{\delta }_{\sigma }{w}^{\ast }),$$

where *δ*_*x*_, *δ*_*y*_, and *δ*_*σ*_ denote the spatial difference operator in horizontal and vertical directions and can be written as

10 $${{\delta }_{x}a|}_{i,j,k}=\frac{{a}_{i+1,j,k}-{a}_{i,j,k}}{\Delta x},\,{{\delta }_{y}a|}_{i,j,k}=\frac{{a}_{i,j+1,k}-{a}_{i,j,k}}{\Delta y},\,{{\delta }_{\sigma }a|}_{i,j,k}=\frac{1}{{D}_{i,j}^{n}}\frac{{a}_{i,j,k+1}-{a}_{i,j,k}}{\Delta {\sigma }_{k}}.$$

${{\mathcal{F}}}_{3}^{n}$ is the sum of advection and horizontal diffusion terms in *z* direction. *q*^*n*-1/2^ denotes the non-hydrostatic pressure *q* at time level *n*-1/2, which is less than the time level of the momentum variables by half a time step size for the FB difference scheme. D^*^ = H + η^*^. The implicit scheme is used in discretizing the vertical diffusion terms (the third terms in right hand side of Eq. (7~9)), so strict limitations on the time step can be avoided. A linear equation system with a tridiagonal coefficient matrix is solved based on the Thomas algorithm to obtain the provisional velocities^36^.

As previous studies introduced^17,27,42^, the calculated provisional velocities should be corrected by the non-hydrostatic pressure increments to obtain the velocities at time level *n* + 1, the expression can be written as

11 $$\frac{{u}_{i,j,k}^{n+1}-{u}_{i,j,k}^{\ast }}{\Delta t}=-\frac{1}{2{\rho }_{0}}({{\delta }_{x}q{\prime} |}_{i,j,k}+{{\delta }_{x}q{\prime} |}_{i-1,j,k}+{{\delta }_{x}\sigma |}_{i,j,k}{\delta }_{\sigma }q{\prime} +{{\delta }_{x}\sigma |}_{i-1,j,k}{\delta }_{\sigma }q{\prime} ),$$

12 $$\frac{{v}_{i,j,k}^{n+1}-{v}_{i,j,k}^{\ast }}{\Delta t}=-\frac{1}{2{\rho }_{0}}({{\delta }_{y}q{\prime} |}_{i,j,k}+{{\delta }_{y}q{\prime} |}_{i,j-1,k}+{{\delta }_{y}\sigma |}_{i,j,k}{\delta }_{\sigma }q{\prime} +{{\delta }_{y}\sigma |}_{i,j-1,k}{\delta }_{\sigma }q{\prime} ),$$

13 $$\frac{{w}_{i,j,k}^{n+1}-{w}_{i,j,k}^{\ast }}{\Delta t}=-\frac{1}{2{\rho }_{0}{D}_{i,j}^{\ast }}({{\delta }_{\sigma }q{\prime} |}_{i,j,k}+{{\delta }_{\sigma }q{\prime} |}_{i,j,k-1}).$$

The temperature and salinity equations are also solved using the FB scheme that is completely similar to momentum equations, with their discretized time steps differing by half a step from those of the momentum equations, that is to say, $\frac{{C}_{i,j,k}^{\ast }{D}_{i,j}^{\ast }-{C}_{i,j,k}^{n+1/2}{D}_{i,j}^{n+1/2}}{\varDelta t}$ and $\frac{{C}_{i,j,k}^{n+3/2}{D}_{i,j}^{n+3/2}-{C}_{i,j,k}^{\ast }{D}_{i,j}^{\ast }}{\varDelta t}$, *C* can be chosen as *T* or *s*.

Based on the continuity equation, the increment of non-hydrostatic pressure *q*′ can be calculated from the Poisson equation as follows:

14 $$\frac{{\partial }^{2}q{\prime} }{\partial {x}^{2}}+\frac{{\partial }^{2}q{\prime} }{\partial {y}^{2}}+\left[{\left(\frac{\partial \sigma }{\partial x}\right)}^{2}+{\left(\frac{\partial \sigma }{\partial y}\right)}^{2}+\frac{1}{{D}^{2}}\right]\frac{{\partial }^{2}q{\prime} }{\partial {\sigma }^{2}}=\frac{{\rho }_{0}}{2\Delta t}\left(\frac{\partial {u}^{\ast }}{\partial x}+\frac{\partial \sigma }{\partial x}\frac{\partial {u}^{\ast }}{\partial \sigma }+\frac{\partial {v}^{\ast }}{\partial y}+\frac{\partial \sigma }{\partial y}\frac{\partial {v}^{\ast }}{\partial \sigma }+\frac{1}{D}\frac{\partial {w}^{\ast }}{\partial \sigma }\right),$$

where the provisional velocities u^*^, v^*^ and w^*^ are calculated from Eqs. (11~13). Like other tracer variables in the Arakawa-C grid system, the Poisson Eq. (14) can be discretized as

15 $$\begin{array}{l}\frac{1}{4}\{{{\delta }_{x}[{{\delta }_{x}q{\prime} |}_{i,j,k}+{{\delta }_{x}q{\prime} |}_{i-1,j,k}]|}_{i,j,k}+{{\delta }_{x}[{{\delta }_{x}q{\prime} |}_{i,j,k}+{{\delta }_{x}q{\prime} |}_{i-1,j,k}]|}_{i-1,j,k}\}+\\ \frac{1}{4}\{{{\delta }_{y}[{{\delta }_{y}q{\prime} |}_{i,j,k}+{{\delta }_{y}q{\prime} |}_{i,j-1,k}]|}_{i,j,k}+{{\delta }_{y}[{{\delta }_{y}q{\prime} |}_{i,j,k}+{{\delta }_{y}q{\prime} |}_{i,j-1,k}]|}_{i,j-1,k}\}+\\ \left\{\begin{array}{c}\frac{1}{4}\{{{\delta }_{x}[{{\delta }_{x}\sigma |}_{i,j,k}+{{\delta }_{x}\sigma |}_{i-1,j,k}]|}_{i,j,k}+{{\delta }_{x}[{{\delta }_{x}\sigma |}_{i,j,k}+{{\delta }_{x}\sigma |}_{i-1,j,k}]|}_{i-1,j,k}\}+\\ \frac{1}{4}\{{{\delta }_{y}[{{\delta }_{y}\sigma |}_{i,j,k}+{{\delta }_{y}\sigma |}_{i,j-1,k}]|}_{i,j,k}+{{\delta }_{y}[{{\delta }_{y}\sigma |}_{i,j,k}+{{\delta }_{y}\sigma |}_{i,j-1,k}]|}_{i,j-1,k}\}+\frac{1}{{D}_{i,j}^{2}}\end{array}\right\}\cdot \\ \frac{1}{4}\{{{\delta }_{\sigma }[{{\delta }_{\sigma }q{\prime} |}_{i,j,k}+{{\delta }_{\sigma }q{\prime} |}_{i,j,k-1}]|}_{i,j,k}+{{\delta }_{\sigma }[{{\delta }_{\sigma }q{\prime} |}_{i,j,k}+{{\delta }_{\sigma }q{\prime} |}_{i,j,k-1}]|}_{i,j,k-1}\}=\\ \frac{{\rho }_{0}}{2\Delta t}\left[\begin{array}{c}{{\delta }_{x}{u}^{\ast }|}_{i,j,k}+\frac{1}{2}({{\delta }_{x}\sigma |}_{i,j,k}{{\delta }_{\sigma }{u}^{\ast }|}_{i,j,k}+{{\delta }_{x}\sigma |}_{i-1,j,k}{{\delta }_{\sigma }{u}^{\ast }|}_{i,j,k})+\\ {{\delta }_{y}{v}^{\ast }|}_{i,j,k}+\frac{1}{2}({{\delta }_{y}\sigma |}_{i,j,k}{{\delta }_{\sigma }{v}^{\ast }|}_{i,j,k}+{{\delta }_{y}\sigma |}_{i,j-1,k}{{\delta }_{\sigma }{v}^{\ast }|}_{i,j,k})+\\ \frac{1}{2}({{\delta }_{\sigma }{w}^{\ast }|}_{i,j,k}+{{\delta }_{\sigma }{w}^{\ast }|}_{i,j,k-1})\end{array}\right]\end{array}$$

The linear equation system (15) with three-dimensional tri-diagonally dominant coefficient matrix can be solved using some mature numerical algorithms, such as the generalized minimum residual method (GMRES), direct quasi-GMRES (DQGMRES) with QR factorization preconditioning, flexible-GMRES (FGMRES), and so on^45,46^. Auclair *et al*.^17^ demonstrated that the DQGMRES algorithm with the incomplete LU factorization combined with threshold dropping method (ILUT) is preferable, because the computational costs are relatively low, the calculation accuracy is high, and the solving processes are robust. Once the non-hydrostatic pressure increment *q*′ is calculated, the non-hydrostatic component of pressure at time level *n* + 1/2 (*q*^*n*+1/2^) can be obtained from ${q}^{n-1/2}={q}^{n+1/2}+q{\prime}$.

### Experimental design

The MASNUM-NonHydro OGCM is used for numerical simulation. The model domain covers the NSCS (105° ~ 125° E, 15° ~ 26° N) with a horizontal resolution of (1/90)° × (1/90)° and 72 σ vertical layers (Table 1). The gridded bathymetric data from the global General Bathymetric Chart of the Oceans 2024 (GEBCO_24)^47^ is used to construct the topography for ocean modelling. The GEBCO_24 data can be downloaded from https://www.gebco.net/data-products-gridded-bathymetry-data/gebco2024-grid. The minimum and maximum depths are set to 20 m and 5 000 m, respectively. Fluxes of momentum, heat and freshwater are computed using a bulk formula, and the required parameters are interpolated based on the European Centre for medium-range weather forecasts (ECMWF) reanalysis Version 5 (ERA5) data^48,49^, with the horizontal and time resolutions of (1/4)° × (1/4)° and 1 h, respectively. The ERA5 data are available at the Climate Data Store (CDS) implemented by ECMWF (https://cds.climate.copernicus.eu/datasets/reanalysis-era5-single-levels?tab = download). Once users have successfully registered, stated their purpose, and passed the review process, they gain free access to download all the ERA5 data. The velocity, temperature, and salinity fields, along with daily lateral boundary conditions, are from the China ocean reanalysis data Version 2.0 (CORA2)^50^ produced by the National Marine Data and Information Service (NMDIS), with a horizontal resolution of (1/12)° × (1/12)° and daily updates. The CORA2 data can be downloaded from https://mds.nmdis.org.cn/pages/dataViewDetail.html?dataSetId=a3b8d3c79a7543cbb8680ad93219fa95. The tidal forcing of eight main tidal constituents, including M2, S2, N2, K2, K1, O1, P1, and Q1, is also added at the open lateral boundaries using the Flather method^51^. The tidal sea level and current data interpolated from the Tidal Predictions in the Xtended Oregon state (TPXO) dataset^52^ are used for tidal forcing. The TPXO data can be downloaded from https://www.tpxo.net/tpxo-products-and-registration.

**Table 1:** Vertical coordinates for each σ-layer and Z-level.

No.	σ	Z (m)	No.	σ	Z (m)	No.	σ	Z (m)
1	−1.0000	0	26	−0.6212	−160	51	−0.2424	−800
2	−0.9848	−2	27	−0.6061	−170	52	−0.2273	−850
3	−0.9697	−4	28	−0.5909	−180	53	−0.2121	−900
4	−0.9545	−6	29	−0.5758	−190	54	−0.1970	−950
5	−0.9394	−8	30	−0.5606	−200	55	−0.1818	−1000
6	−0.9242	−10	31	−0.5455	−220	56	−0.1667	−1100
7	−0.9091	−12	32	−0.5303	−240	57	−0.1515	−1200
8	−0.8939	−14	33	−0.5152	−260	58	−0.1364	−1300
9	−0.8788	−16	34	−0.5000	−280	59	−0.1212	−1400
10	−0.8636	−18	35	−0.4848	−300	60	−0.1061	−1500
11	−0.8485	−20	36	−0.4697	−320	61	−0.0909	−1600
12	−0.8333	−25	37	−0.4545	−340	62	−0.0758	−1700
13	−0.8182	−30	38	−0.4394	−360	63	−0.0606	−1800
14	−0.8030	−40	39	−0.4242	−380	64	−0.0455	−1900
15	−0.7879	−50	40	−0.4091	−400	65	−0.0303	−2000
16	−0.7727	−60	41	−0.3939	−420	66	−0.0152	−2200
17	−0.7576	−70	42	−0.3788	−440	67	−0.0076	−2400
18	−0.7424	−80	43	−0.3636	−460	68	−0.0038	−2600
19	−0.7273	−90	44	−0.3485	−480	69	−0.0019	−2800
20	−0.7121	−100	45	−0.3333	−500	70	−0.0010	−3000
21	−0.6970	−110	46	−0.3182	−550	71	−0.0008	−3200
22	−0.6818	−120	47	−0.3030	−600	72	0.0000	−3400
23	−0.6667	−130	48	−0.2879	−650	73		−3600
24	−0.6515	−140	49	−0.2727	−700	74		−3800
25	−0.6364	−150	50	−0.2576	−750	75		−4000

The K-profile parameterization scheme (KPP)^53^ is adopted to describe the impact of vertical mixing. In this scheme, the turbulent viscosity and diffusivity are computed based on the Richardson number and boundary layer depth. The nonlinear equation of state (EOS)^54^ is used to compute the *in-situ* density as a function of potential temperature, salinity and pressure. The bottom momentum stress is calculated using a parameterization scheme based on the velocity, thickness of the σ layer, and friction coefficient at the bottom to preserve the bottom velocity direction for stability^55^. To ensure stability, the time steps of the barotropic and baroclinic modes are set to 0.4 s and 2 s, respectively. The high-resolution OGCM is integrated for 3 months (from October1, 2018, to December 31, 2018) to achieve a stable state. The datasets in this study are derived from model results in 2019. In this study, we designed another experiment based on the hydrostatic MASNUM OGCM (MASNUM-Hydro) with identical configurations to the MASNUM-NonHydro setup for dataset validation purposes.

## Data Records

### Format of data

This dataset includes the model outputs, which contain three two-dimensional (2D) and five three-dimensional (3D) ocean state variables (Table 2). The horizontal and temporal resolutions are (1/90)° × (1/90)° and daily, respectively. All model outputs are linearly interpolated vertically from σ layers to 75 fixed-depth (Z) levels (Table 1). These Z levels are determined based on the distribution of dynamic characteristics and divided into four vertical zones: surface region (0–200 m), main thermocline region (200–500 m), intermediate region (500–2000 m), and deep region (2000–4000 m). The number of levels in each zone are 24, 21, 18, and 12, with thicknesses ranging from 2–10 m, 20–50 m, 80–150 m, and 200–300 m, respectively.

**Table 2:** Descriptions of data variables.

Dimension	Variable name	Long name	Unit	Horizontal Grid^1^
2	Zeta	sea surface height	m	t-grid
	ubar	barotropic sea water velocity in x (zonal) direction	m/s	u-grid
	vbar	barotropic sea water velocity in y (meridional) direction	m/s	v-grid
3	temp	potential temperature	°C	t-grid
	salt	salinity	0.001	t-grid
	u	sea water velocity in x (zonal) direction	m/s	u-grid
	v	sea water velocity in y (meridional) direction	m/s	v-grid
	w	sea water velocity in z (depth) direction	m/s	t-grid

^1^Arakawa-C grid is used for model calculations, the horizontal velocity variables and tracer variables (temperature and salinity) are staggered in spatial distribution, tracer variables are located in the center of the grids (t-grid), while x-direction/y-direction velocity variables are located in the boundary of the grids (u-grid or v-grid).

### Access to the data

All model outputs are stored in the netCDF-4 classic format and archived in the Science Data Bank (10.57760/sciencedb.27570)^56^. The total data size is approximately 290 GB.

## Technical Validation

To evaluate the dataset’s ability to characterize the dynamic features of the NSCS, three aspects of comparison are presented in the study: ITs, temperature profiles, and SST. An idealized standing wave experiment is also designed to assess the advantages of non-hydrostatic calculations in high-resolution simulations. The dataset is constructed based on the outputs from MASNUM-NonHydro, and MASNUM-Hydro results are also used for comparison.

### Idealized case: standing wave in a closed basin

This experiment is designed to test the effects of the non-hydrostatic calculations on the simulation accuracy. The simulation of the small amplitude standing waves with closed boundaries provides an appropriate example for testing the energy conservation and flow structure simulated by non-hydrostatic OGCMs^57,58^. This “standing wave” test case is constructed in a two-dimensional closed square basin with 10 m depth (*D*) and 10 m width (*L*), which is in *x*-*z* Cartesian coordinate system. The initial surface elevation is expressed as

16 $$\eta (x)={\eta }_{0}\,\cos (kx),$$

where *k* = 2π/*nL* is the wave number, *n* is set to 2 for standing waves. *η*_0_ is chosen as 0.1 m which is 1% of the depth *D*, making the small amplitude wave theory applicable. Initial velocities are set to zero, and there is no friction on the lateral and bottom boundaries. The sea water in the closed basin is assumed to be inviscid and irrotational, so the waves will propagate along the *x* axis, and the governing equations can be written as

17 $$\frac{\partial u}{\partial t}=-\frac{1}{{\rho }_{0}}\frac{\partial p}{\partial x},$$

18 $$\frac{\partial w}{\partial t}=-\frac{1}{{\rho }_{0}}\frac{\partial p}{\partial z},$$

19 $$\frac{\partial u}{\partial x}+\frac{\partial w}{\partial z}=0,$$

where $p={\rho }_{0}g\eta +q$ is the sum of the hydrostatic pressure related to the surface elevation and nonhydrostatic pressure *q*. *ρ*_0_ and *g* are set to 1000 kg/m^3^ and 9.8 m/s^2^, respectively. The analytical solution can be derived based on the small amplitude wave theory^59,60^, and the velocities and surface elevation can be calculated directly as

20 $$\eta (x,t)={\eta }_{0}\,\cos (kx)\cos (\omega t),$$

21 $$u(x,z,t)=\omega {\eta }_{0}\frac{\cosh [k(z+H)]}{\sinh (kH)}\sin (kx)\sin (\omega t),$$

22 $$w(x,z,t)=-\omega {\eta }_{0}\frac{\sinh [k(z+H)]}{\sinh (kH)}\cos (kx)\sin (\omega t),$$

where $\omega =\sqrt{gk\,\tanh (kH)}$ is the wave frequency.

**Fig. 2:**
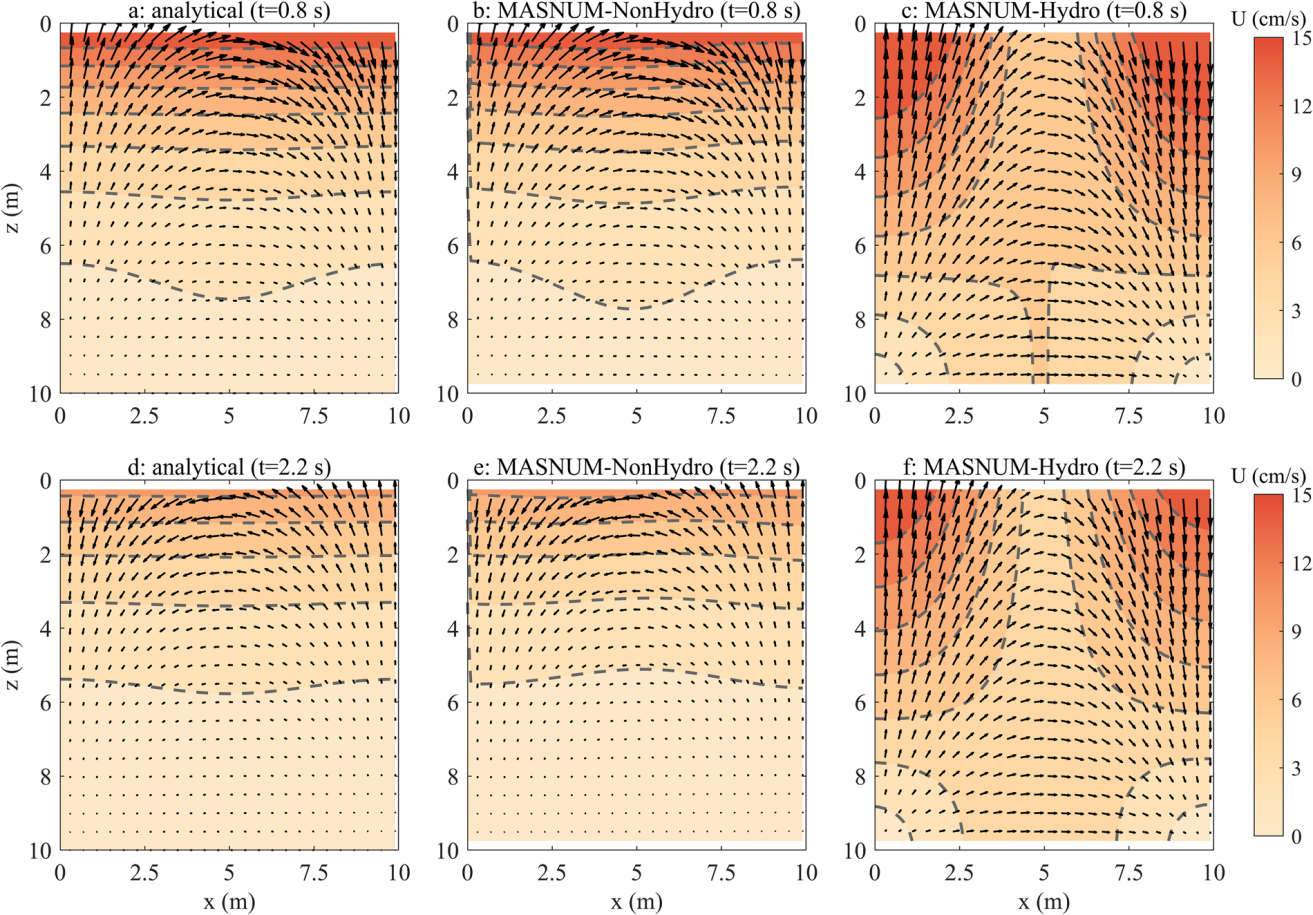
Sections of analytical results (**a** and **d**), numerical simulation by MASNUM-NonHydro (**b** and **e**) and MASNUM-Hydro (**c** and **f**) for the current vectors and velocity magnitudes (gray dashed contours with colored shades) (unit: cm/s) in the *x*-*z* plane at t = 0.8 and 2.2 s.

The standing waves are simulated by the MASNUM OGCM with a uniform grid spacing of 0.2 m in the *x* and *z* directions. The time step size is set to 0.1 s. This case enables the examination of the stability and accuracy of the numerical algorithm, and compares the physical mechanisms of OGCMs with hydrostatic and non-hydrostatic pressure. It can also evaluate numerical damping and mass conservation of the calculation, since the fluid oscillates periodically in the closed domain^21^. Figure 2 shows the current structure in the *x*-*z* plane calculated analytically and simulated by MASNUM-Hydro and MASNUM-NonHydro at 0.8 and 2.2 s, respectively. Overall, both simulated velocity vectors and velocity magnitudes by MASNUM-NonHydro are more closely aligned with the analytical results than those by MASNUM-Hydro. The velocity magnitudes are largest at the surface and decrease gradually with depth in the whole region. For MASNUM-Hydro, the simulated horizontal velocities in the middle region (*x* = 4 m to 6 m) are too weak, causing fluid to accumulate on both sides and resulting in larger vertical velocities. It indicates that the simulated hydrostatic pressure gradient is inaccurate in MASNUM-Hydro. The absolute mean deviations over the entire region of the velocity decrease from 0.0367 m/s and 0.0385 m/s, for MASNUM-Hydro, to 0.0021 m/s and 0.0026 m/s, for MASNUM-NonHydro, representing reductions of 94.3% and 93.4%, respectively.

**Fig. 3:**
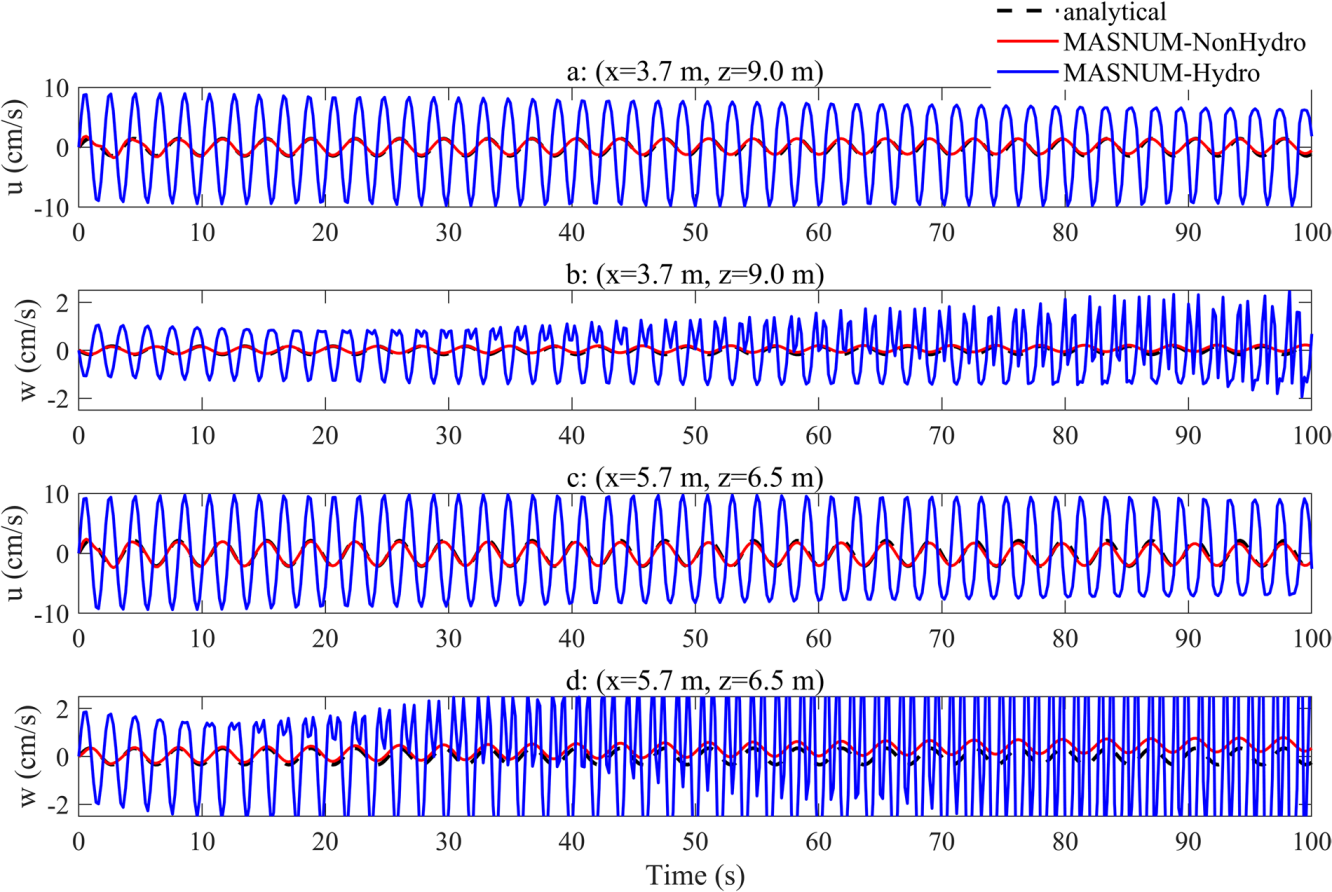
Time series of the u-components and w-components of the velocity from analytical results (black dashed curves), MASNUM-NonHydro (red curves) and MASNUM-Hydro (blue curves) models at the locations of *x* = 3.7 m, *z* = 9.0 m (a and b), and *x* = 5.7 m, *z* = 6.5 m (c and d) over 100 s integration.

The time variation of the velocity *u* and *w* at two locations (*x* = 3.7 m, *z* = 9.0 m, and *x* = 5.7 m, *z* = 6.5 m) over 100 s integration is shown in Fig. 3. In Fig. 3a,c, the red curves, representing *u* simulated by MASNUM-NonHydro, and the black curves, representing the analytical results, nearly overlap, indicating that the simulated *u* for MASNUM-NonHydro is in close agreement with the analytical results during the entire period. Meanwhile, the differences between the simulated *w* for MASNUM-NonHydro and the analytical results are significantly smaller than those for MASNUM-Hydro (Fig. 3b,d). This suggests that the MASNUM-NonHydro model effectively captures the long-term variation of the current field. In contrast, the oscillation amplitudes of the simulated *u* and *w* by MASNUM-Hydro gradually increase over time and are significantly larger than the analytical results. Additionally, the crests and troughs of the simulated *u* and *w* by MASNUM-Hydro indicate that the oscillation periods are quite different from the analytical results. Based on the dispersion relation, the wave period in this case can be estimated as 3.6 s from the wave frequency, but the wave period is as small as 2.0 s based on the hydrostatic assumption^57–59^.

**Fig. 4:**
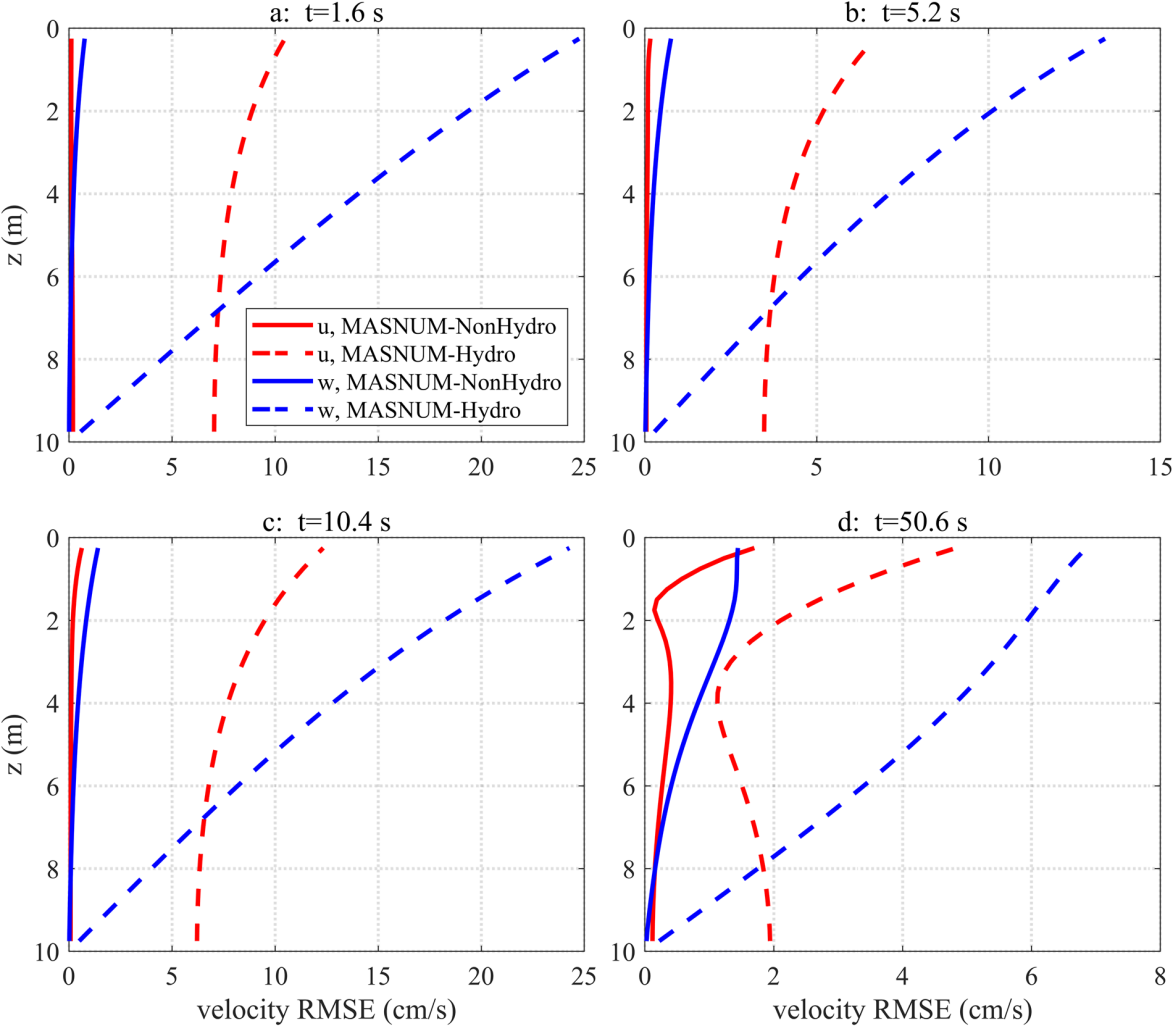
The RMSEs of the velocity *u* (red) and *w* (blue) between the simulation by MASNUM-NonHydro (solid lines) and MASNUM-Hydro (dashed lines) at different depths at t = 1.6 s, 5.2 s, 10.4 s, and 50.6 s.

To evaluate the deviation between analytical results and numerical simulations at different depths, the root mean square errors (RMSEs) of the velocity *u* and *w* at t = 1.6 s, 5.2 s, 10.4 s, and 50.6 s are calculated (Fig. 4). It can be seen that the RMSEs for MASNUM-Hydro are larger than those of MASNUM-NonHydro especially near the surface. This is because the maximum velocities occur at the surface, with velocities decreasing with depth and nearly reaching zero at the bottom. The RMSEs for simulated *u* and *w* by MASNUM-Hydro are as large as 25 cm/s and over 10 cm/s, respectively. The reason is that the oscillation periods for the MASNUM-Hydro simulation differ significantly from the analytical results, and the inconsistent variation leads to these large RMSEs. For MASNUM-NonHydro, RMSEs increase slightly over time and remain below 2 cm/s at all depths at t = 50.6 s. Improvements to the numerical algorithms for the MASNUM model will be made in future works to reduce finite difference calculation errors and further decrease the RMSEs for MASNUM-NonHydro.

### Scale analysis of (1/90)° simulations

**Fig. 5:**
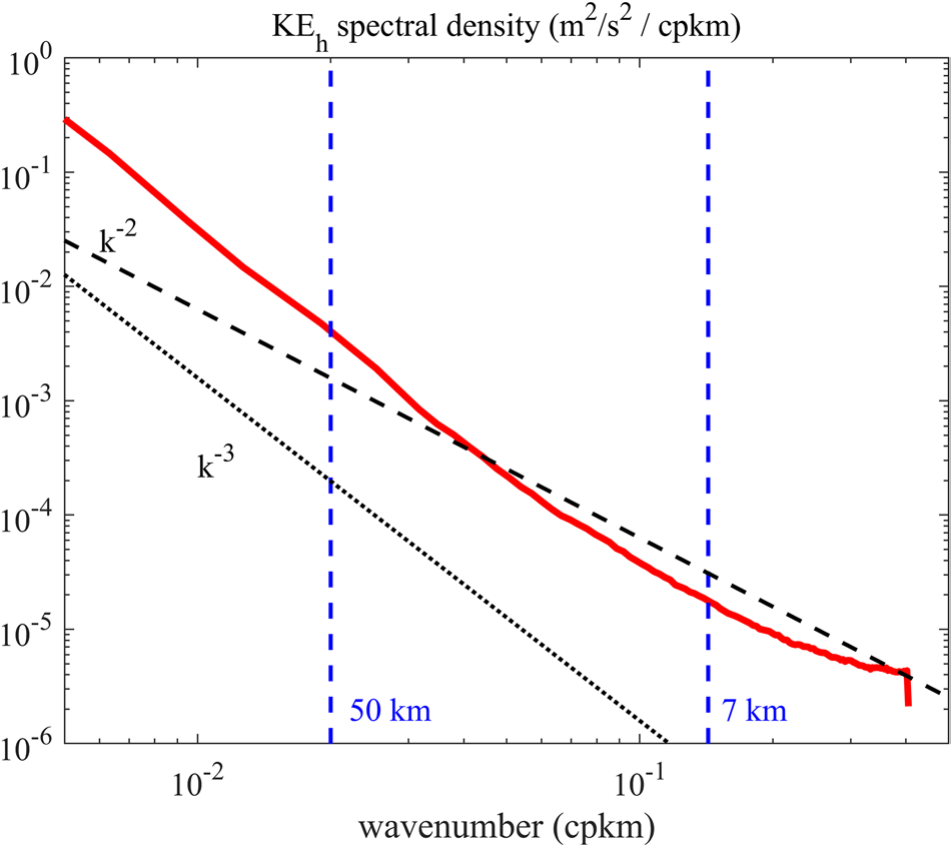
Spatially averaged horizontal wavenumber spectra of horizontal kinetic energy (KE_h_) at the upper 100 m layer in the NSCS.

Previous studies demonstrated that submesoscale processes are ubiquitous throughout the NSCS^2,13,15^. The submesoscales are characterized by *Ο*(1) gradient Rossby number, $\mathrm{Ro}\,=\,|\zeta |/f$, which means that inertial and Coriolis forces both play a significant and competing role in the dynamics of the flow, where $\zeta ={\partial }_{x}v-{\partial }_{y}u$ is the vertical relative vorticity, *u* and *v* are the horizontal velocities, *f* denotes the planetary vorticity, *x* and *y* are the components of Cartesian coordinate. The spatially averaged horizontal wavenumber spectrum of the horizontal kinetic energy in the NSCS (Fig. 5) indicates the minimum wavelength that the model can resolve. The slope of spectrum is close to *k*^−2^ when the wavelength is less than 50 km, indicating the dominant effects of the ageostrophic processes according to the quasi- geostrophic theory. A transitioning point corresponding to wavelength of 7 km, where the spectrum transits from a steady state to fluctuation, can be regarded as the minimum resolvable scale. This scale is approximately 4–5 times the grid spacing for OGCMs. Notably, although the model in this study can capture the motions at scales of approximately 7 km, it is indeed not able to fully resolve all the submesoscale processes. Further increasing the resolution and optimizing the related parameterization schemes is a feasible approach for advancing the study of submesoscale dynamics.

### The dynamic patterns of internal tides

At the LS, ITs are generated periodically when barotropic tides flow over double ridges. They propagate into the NSCS, accompanying interaction with other multi-scale processes and the continental shelf^61,62^. The simulated vertical velocities and surface elevation are validated by comparing with the MODIS images, which are currently a key method for observing ITs because the information of the ITs can be extracted obviously as some strips of alternating light and dark in the MODIS images^63,64^. Artificial intelligence techniques have been applied to geoscience research and proven to be useful and valuable for the IT detection from the remote sensing images^65–68^. Based on the deep convolutional neural networks (DCNN), Zhang *et al*.^69^ proposed a specially tailored IT (or internal waves) extraction network (IWE-Net) with high accuracy and robustness for extracting the IT signature. These methods greatly promote the IT detection and related research using remote sensing data and are of great significance for applying intelligent techniques. In this study, three MODIS images are used to validate the simulated ability of OGCMs, and the IWE-Net method is adopted to extract the wave crest locations of the ITs.

**Fig. 6:**
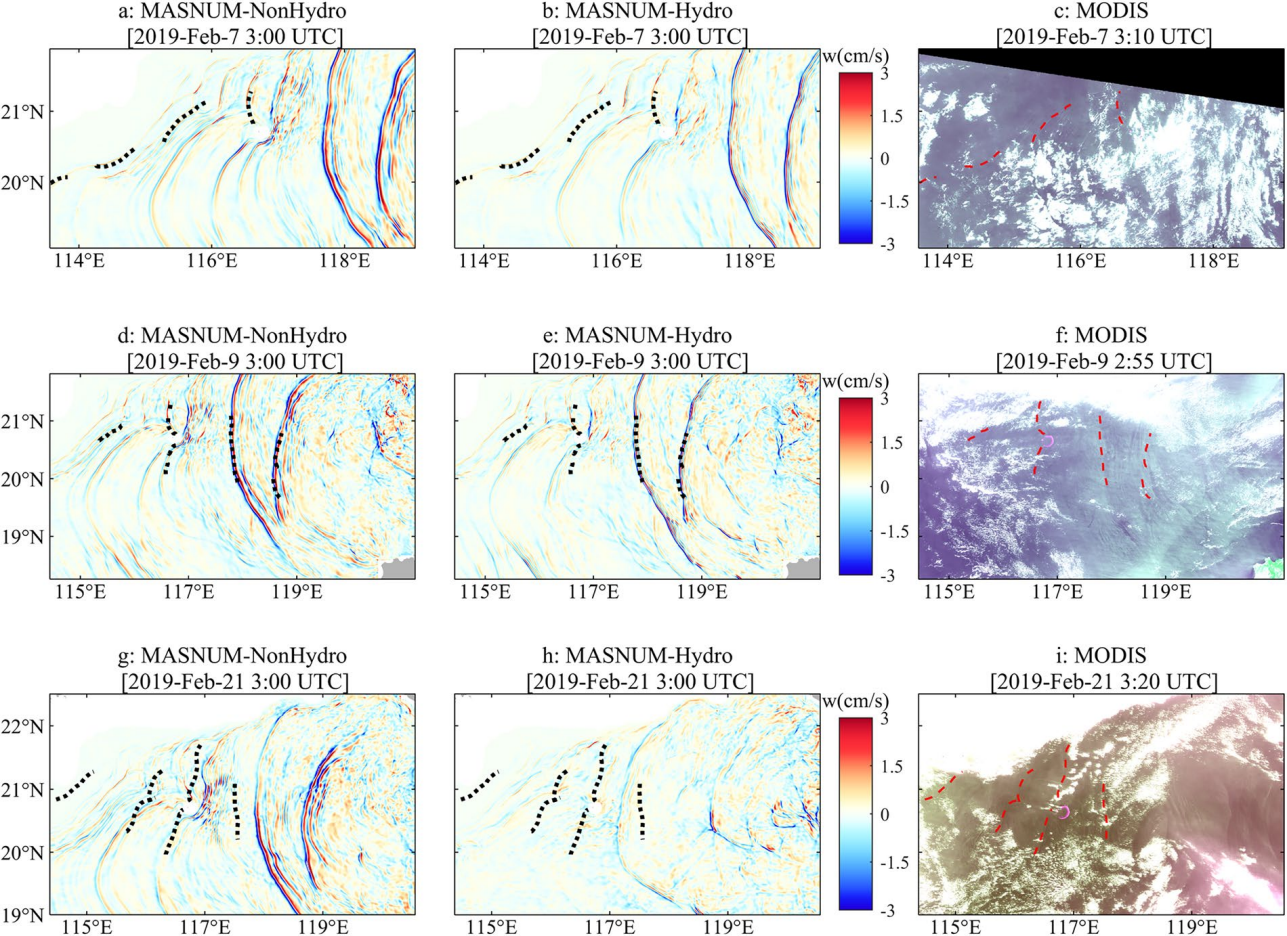
The simulated vertical velocity at the depth of 80 m at 3:00 UTC on February 7, 2019 (**a** and **b**), 3:00 UTC on February 9, 2019 (**d** and **e**), and 3:00 UTC on February 21, 2019 (**g** and **h**). The MASNUM-NonHydro (**a,** **d** and **g**) and MASNUM-Hydro (**b,** **e** and **h**) are used for IT simulation. The MODIS images obtained at 3:10 UTC on February 7, 2019 (**c**), 2:55 UTC on February 9, 2019 (**f**), and 3:20 UTC on February 21, 2019 (**i**) are also shown. Red dashed curves represent the wave crest locations extracted from the MODIS images using the IWE-Net method, which are overlayed to the vertical velocity simulation (**a,b,d,e,g** and **h**) as black dotted curves.

The simulated vertical velocity at a depth of 80 m is compared with the MODIS images (Fig. 6). The high-value band distribution of the vertical velocity reflects the IT patterns, which are compared with the wave crest locations extracted from the images. The time for the model results is chosen as the one closest to the observing time of the MODIS images, because the model results are only stored hourly due to the huge amount of data. It is noted that some of the ITs are not obvious and even not observed in the MODIS images due to the cloud cover. Both the wave crest locations and IT patterns simulated by MASNUM-NonHydro and MASNUM-Hydro are generally consistent with those detected in the images (Fig. 6). The simulated vertical velocity fields indicate that the ITs propagate as wave packets in different directions, which also occur in the MODIS images. Compared to MASNUM-Hydro, MASNUM-NonHydro simulates stronger vertical velocity, especially in the high-value regions, revealing more small-scale processes. This implies that the simulated vertical velocity (or the IT processes) will be enhanced when the non-hydrostatic pressure is incorporated into OGCMs. Furthermore, finer structures can be found in the MASNUM-NonHydro simulations. However, it is worth noting that ITs in the NSCS exhibit strong and distinct signals, which can be depicted successfully by both hydrostatic or non-hydrostatic models, and this comparison alone cannot confirm that MASNUM-NonHydro offers higher accuracy for IT simulation, because only wave crest locations in sea surface layers are extracted from the MODIS images. Further evaluation using *in-situ* and remote sensing data will provide a more comprehensive assessment of the model’s performance.

**Fig. 7:**
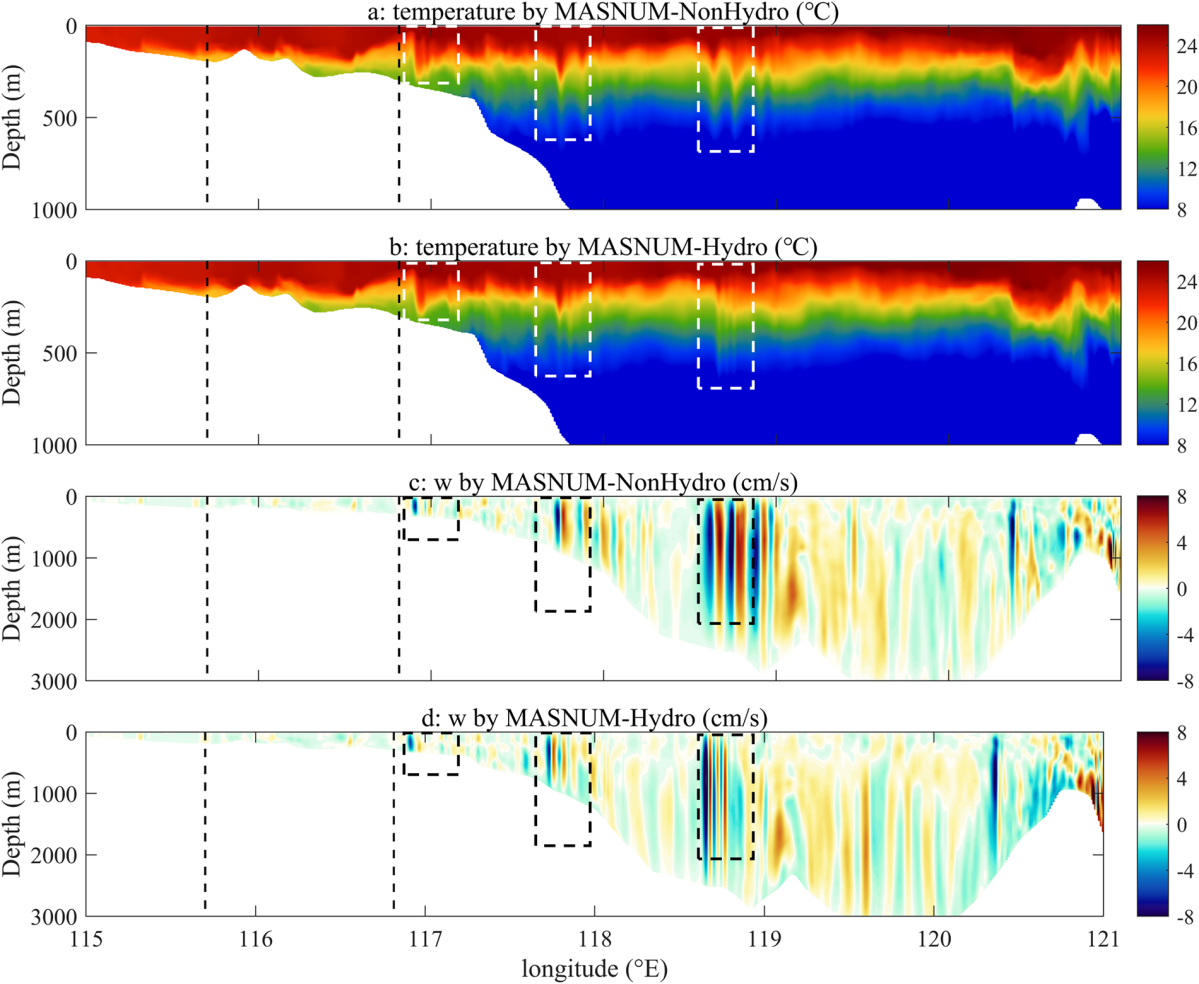
The temperature (**a** and **b**) and vertical current (**c** and **d**) structure along the 21°N section at 3:00 UTC on February7, 2019. Black dashed lines represent the wave crest locations extracted from the MODIS images shown in Fig. 6a,c. White dashed boxes in (**a**) and (**b**) and black dashed boxes in (**c**) and (**d**) correspond to the regions filled with IT signals.

The section along 21°N, which is in the IT propagation direction at 3:00 UTC on February 7, 2019, is selected to illustrate the vertical structure of temperature and vertical velocity for clarifying the effects of non-hydrostatic pressure (Fig. 7). Figure 7a,b display the temperature structure in the upper 1000 m with obvious fluctuations induced by ITs (white dashed boxes in Fig. 7a,b). The fluctuations corresponded to regions of strong vertical velocity between approximately 118.6° and 118.9° E, approximately 117.7° and 117.9° E, and approximately 116.8° and 117.0° E (black dashed boxes in Fig. 7c,d). The simulation indicates that the ITs originate west of the LS area, with maximum vertical velocities of approximately 8 cm/s. In comparison, the simulated ITs by MASNUM-Hydro are smaller in value and coverage than those from MASNUM-NonHydro.

To further examine the differences between the simulations of MASNUM-Hydro and MASNUM-NonHydro, regionally absolute averaged values of two variables, *w* and *ρ*, are calculated. As two of the most typical diagnostics, *w* and *ρ* can intuitively and clearly characterize the differences between the two models in solving the vertical momentum equation and calculating the pressure, respectively. During the simulation period, the averaged *w* (4.61 × 10^−3^ m/s) and *ρ* (1026.59 kg/m^3^) for MASNUM-NonHydro are larger than those (4.32 × 10^−3^ m/s and 1026.38 kg/m^3^) for MASNUM-Hydro. Maximum value of *w* and *ρ* increase from 5.50 × 10^−3^ m/s (MASNUM-Hydro) to 5.98 × 10^−3^ m/s (MASNUM-NonHydro), from 1026.59 kg/m^3^ (MASNUM-Hydro) to 1026.66 kg/m^3^ (MASNUM-NonHydro). As shown in Eq. (13), the contribution of the introduced non-hydrostatic calculation can be written as $-\frac{1}{2{\rho }_{0}{D}_{i,j}^{\ast }}({{\delta }_{\sigma }q{\prime} |}_{i,j,k}+{{\delta }_{\sigma }q{\prime} |}_{i,j,k-1})$ for the vertical momentum equation. Based on the comparison of the regional averaged *w*, the simulation of *w* will increase by up to approximately 15% after introducing non-hydrostatic calculation. A quantitative comparison of *w* and *ρ* indicates that the non-hydrostatic calculation enhances the vertical flow velocity, thereby intensifying the exchange with deeper cold water and increasing the density.

**Fig. 8:**
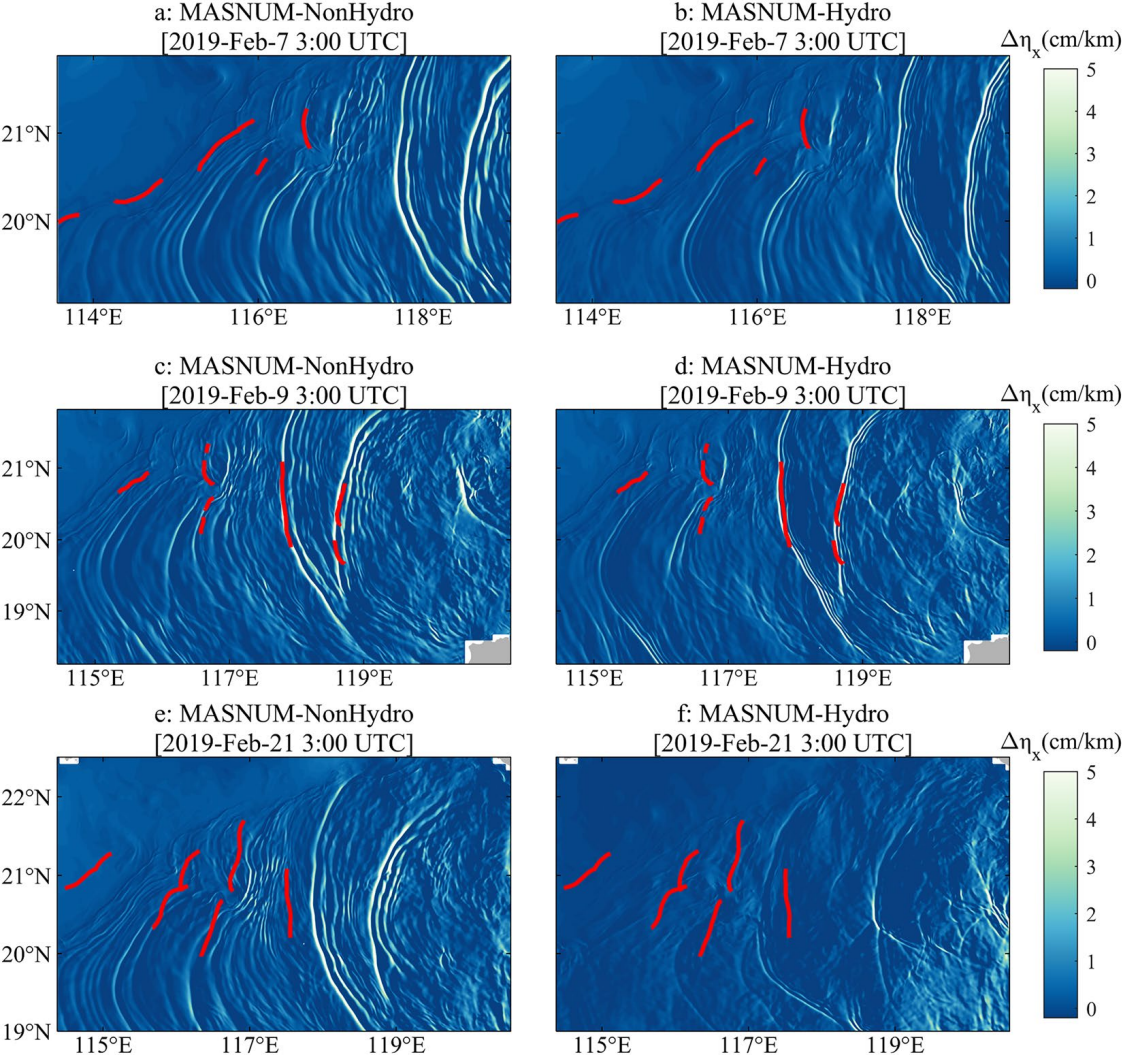
Sea surface height gradients simulated by MASNUM-NonHydro (**a,** **c** and **e**) and MASNUM-Hydro (**b, d** and **f**) at 3:00 UTC on February 7, 2019 (**a** and **b**), 3:00 UTC on February 9, 2019 (**c** and **d**), and 3:00 UTC on February 21, 2019 (**e** and **f**). Red curves represent the wave crest locations extracted from the MODIS images.

The ITs lead to regions of sea surface convergence and divergence, causing variations in sea surface roughness^70^. Figure 8 shows the gradient of the simulated sea surface elevation in the IT propagation direction (westerly in the NSCS), which also reflects the locations of the wave crests or fronts of the ITs. These results are consistent with previous studies^7,71^. The gradient pattern primarily indicates that the model can simulate sea surface height gradient signals induced by ITs, while the larger magnitude for the non-hydrostatic model is related to its representation of more energetic ITs. In the simulation by MASNUM-NonHydro, there are also more small-scale refined structures compared to the MASNUM-Hydro simulation. The surface elevation gradients simulated by MASNUM-NonHydro are larger than those from MASNUM-Hydro, especially at 3:00 UTC on February 21, 2019.

### The vertical distribution of the temperature

**Fig. 9:**
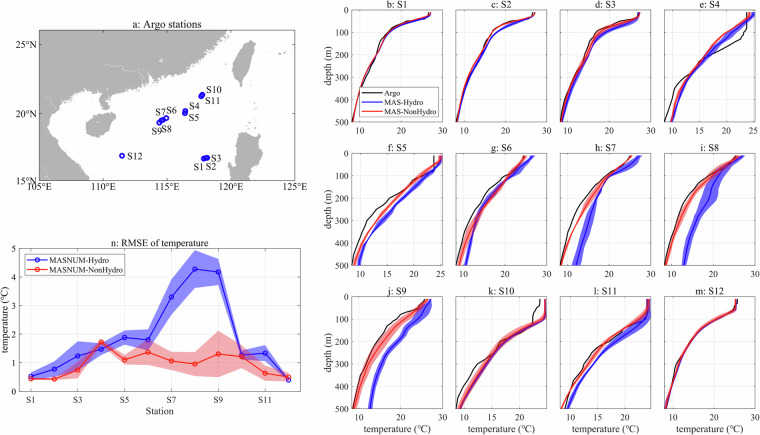
Comparison of the simulated temperature profiles with the Argo data. (**a**): The Argo locations; (**b**)~(**m**): Five simulated temperature profiles closest in time to the Argo observations are selected, the Argo observations (black curves), mean values of the temperature profiles (blue and red curves), and mean values ± standard deviations (blue and red shaded areas) are shown (“MAS-Hydro” and “MAS-NonHydro” are used for short); (**n**): The mean values (lines with circular markers) and mean values ± standard deviations (shaded areas) of the RMSEs between the five temperature profiles and the Argo data.

Twelve Argo profiles in the NSCS were collected during the simulation period to validate the accuracy of the models (Fig. 9), indicating that the vertical distribution of the simulated temperature in the upper 500 m is generally consistent with the Argo data. Spatially, the model results are bilinearly interpolated to the specific locations of the Argo profiles. Temporally, a 5-minute time window is applied, meaning that an Argo data was matched if the observation time differs from the model results by less than 5 minutes. Given that the temperature model outputs are instantaneous three-dimensional fields, while the Argo float used in this study ascended from approximately 1000 meters to the sea surface, observing temperature and depth over an observation period of one to three hours, so the inherent discrepancy between the daily model results and *in-situ* Argo observations induced by temporal mismatch usually arises. Therefore, five simulated temperature profiles closest in time to the Argo data are selected (extending two days forward and backward from the observation date of the Argo data). All temperature profiles were derived by interpolating the corresponding simulated temperature fields at specific times. Figure 9b–m compare the vertical distributions between the mean values and standard deviations of the five temperature profiles and the Argo data. Compared with MASNUM-Hydro, the simulated mean temperature profiles of MASNUM-NonHydro are closer to the observations at nearly all stations, especially at stations S4 to S11, which are located to the west of the LS with a large number of ITs. Moreover, the Argo data lie within the red shaded areas (MASNUM-NonHydro) at a greater number of depth intervals than the blue shaded areas (MASNUM-Hydro). This implies that incorporating non-hydrostatic pressure into OGCMs significantly enhances temperature simulation. The mean values and standard deviations of the RMSEs between the five temperature profiles and the Argo data are also calculated (Fig. 9n). The comparison of the RMSEs also demonstrate that MASNUM-NonHydro achieves higher accuracy, with RMSEs below 1.5 °C at most stations except for stations S4 and S9. At station S4, the upper mixed layer is not well represented owing to insufficient vertical mixing, as it fails to capture the uniform temperature structure within the mixed layer. Improvements by incorporating mixing optimization schemes like wave-induced turbulent mixing, surface wave transport flux residue, and internal-tide-generated turbulent mixing are expected to improve simulation accuracy. At station S9, MASNUM-NonHydro’s RMSEs are much smaller than MASNUM-Hydro’s, though errors may still result from inaccurate topography and background current. The RMSEs of two models exhibit a remarkable difference at stations S6 ~ S9 that are located west of the Dongsha Atoll (DA). A possible explanation for the large difference is that the simulation accuracy of the temperature profiles should be related to the depiction accuracy of ISWs around the DA. A large number of refracted ISWs appear to the west of DA, while many incident ISWs accompanied by relatively fewer reflected ISWs occur to the east of DA^72^. Compared with incident and reflected ISW, refracted ISW typically exhibit larger amplitudes and slower propagation speeds. When the ISW arrives, the upper-ocean vertical velocity is directly enhanced, and its effects can penetrate deep layers, as evidenced by observations from the NSCS showing strong vertical flow and turbulent mixing at 1 500-m layer^73^. This may also lead to larger standard deviations of RMSEs at S6 ~ S9 compared to other stations. Therefore, compared with MASNUM-Hydro, MASNUM-NonHydro is able to capture the stronger vertical motions induced by IGWs around the DA, which enhances the mixing of surface water with colder water from deeper layers, and makes the temperature profile cooler and closer to the Argo data.

### The horizontal distribution of sea surface temperature (SST)

**Fig. 10:**
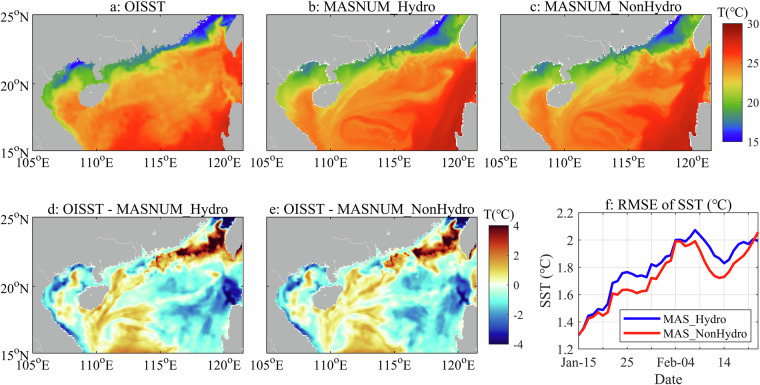
Comparison of the simulated SST and the OISST remote sensing data. (**a**) ~ (**c**): distribution of the OISST and the simulated SST on January 25, 2019; (**d**) and (**e**): the difference that OISST minus the simulated SST; (**f**): the RMSEs of the SST from January 15 to February 28, 2019 (“MAS_Hydro” and “MAS_NonHydro” are used for short).

This study also assesses the impact of non-hydrostatic pressure on SST simulation (Fig. 10), as enhanced vertical processes can induce heat variations and exchanges in the sea surface. The validation uses the daily OISST with a horizontal resolution of (1/4)° × (1/4)°. Figure 10a–e show that both simulated SST distribution patterns by MASNUM-NonHydro and MASNUM-Hydro are similar to the OISST data, with the higher resolution cap capturing more small-scale structure. A colder SST region appears south of the NSCS between about 15° and 17° N, likely due to inaccurate open boundary conditions. Another significant difference is in the Taiwan Strait and its southern area, which is attributed to the inaccuracy of the ERA5 data used in the OGCMs. Figure 10f shows that the RMSEs of SST from MASNUM-NonHydro are less than those from MASNUM-Hydro from January 15 to February 28, 2019, indicating that the simulated SST can be improved significantly by incorporating the non-hydrostatic pressure. It should be noted that the maximum difference in the RMSE between the two simulations is only 0.2 °C approximately, and the differences are more prevalent in regions with larger RMSEs. As a result, the distinction between Fig. 10d,e is indeed not significant visually, implying a relatively weak impact on the sea surface of the non-hydrostatic calculations.

## Data Availability

The dataset of model outputs is freely available without restriction from the Science Data Bank at 10.57760/sciencedb.27570.

## Code availability

The model code and data can be found on Zenodo at 10.5281/zenodo.13689857^74^. An identical version of the model code and data is also available for download on GitHub at https://github.com/jumpen/Non-hydrostatic-MASNUM-Ocean-General-Circulation-Model-codes-and-results.
